# Decreased radiation exposure using pulsed fluoroscopy and a detachable pedicle marker and probe to place pedicle screws: a comparison to current fluoroscopy techniques and CT navigation

**DOI:** 10.1007/s43390-020-00086-5

**Published:** 2020-02-24

**Authors:** Rex A. W. Marco, Michael C. Curry, Faruk Mujezinovic, Judith Linton

**Affiliations:** 1 Shriners Hospitals for Children-Houston, 6977 Main St., Houston, TX 77030 USA; 2 Houston Methodist Orthopedics and Sports Medicine, 6445 Main St, Outpatient Center, Suite 2500, Houston, TX 77030 USA; 3 Joseph Barnhart Department of Orthopedic Surgery, Baylor College of Medicine, 7200 Cambridge, Suite 10A, Houston, TX 77030 USA

**Keywords:** Detachable pedicle marker and probe, Intraoperative fluoroscopy, Radiation exposure, Pedicle screw placement, Scoliosis, Spinal deformity

## Abstract

**Study design:**

Quality improvement evaluation with retrospective analysis.

**Objectives:**

To compare a technique to place pedicle screws (PS) using a novel detachable pedicle marker and probe (DPMP) and pulsed fluoroscopy (PF) vs. conventional technique utilizing PF with standard instruments (SI) and O-arm.

**Summary of background data:**

Spinal fusion with pedicle screw instrumentation (PSI) is the mainstay in treatment of spinal deformities. Reports suggest that CT navigated (O-arm) PS placement is more accurate than fluoroscopy. However, these studies have not considered the increased radiation exposure associated with CT.

**Methods:**

Thirty-six patients with spinal deformity had PSI using PF and DPMPs. Accuracy of PS placement and radiation data from 14 dosimeters placed on the patient and around the operating room was analyzed. Results were compared to published data.

**Results:**

Mean fluoroscopic time was 13.4 s (range 6.0–32.4), and the mean cumulative dose was 3.1 mGy (range 0.2–16.4). Median estimated effective dose to the patient was 0.22 mSv (range 0.0–0.7). The effective dose of radiation was reduced by 80% (0.22 mSv vs. 1.11 mSv) compared to low-dose O-arm. The surgical team did not receive any detectable radiation. The seconds of PF used to assist and confirm placement of PSs was reduced to 1.2 s/level compared to previous reports of 4.49 s/level using SIs. DPMPs reduced fluoroscopy to 0.84 s/PS compared to 7.36 s/PS using SIs to assist and confirm PS placement. PSs were accurately placed in 561 of 576 (97.4%), which is comparable to O-arm and fluoroscopy with SIs.

**Conclusions:**

PS placement using PF and DPMPs to assist and confirm PS placement lowers radiation exposure to the patient and surgical team without compromising accuracy compared to O-arm and fluoroscopy with SIs.

**Level of evidence:**

Therapeutic, Level IV (Retrospective case series, historical control).

## Introduction

Spinal fusion with pedicles screw (PS) instrumentation is widely used to treat patients with scoliosis [1]. Rotational deformities and varying sizes of pedicles make accurate PS placement challenging in terms of avoiding neurological injury and ensuring biomechanical stability of the construct. Imaging modalities are an integral part of PS placement to ensure accuracy.

The use of O-arm for PS placement has become popularized as an alternative to freehand placement with fluoroscopy. Multiple reports suggested that the O-arm significantly reduces the pedicle cortex perforation rate [2–5]. An increasing number of institutions are, thus, mandating the use of O-arm to assist with PS placement in children with spinal deformities. However, studies fail to report the radiation doses ultimately delivered to the patient and OR staff. It is well established that exposure to electromagnetic radiation places patients and the OR team at risk for serious side effects [6–8]. While it is paramount to place PSs accurately, it is also important to reduce radiation exposure to patients, surgeons and staff.

We developed a technique that synthesizes various described principles to maximally reduce radiation and maintain accuracy of PS placement. This study describes a technique using a detachable pedicle marker and probe (DPMP) and pulsed fluoroscopy (PF) to provide a safe and accurate method for placing PSs. To validate this new technique, accuracy of PS placement and cumulative radiation doses in a consecutive series of patients with spinal deformity were compared to patients reported in the literature in which standard instruments (SIs) and either O-arm or PF was used to assist and confirm PS placement.

## Materials and methods

After IRB approval, records for 48 consecutive patients who underwent spinal deformity surgery between December 2012 and March 2015 at either Shriners Hospitals for Children (Sub-group A) or Children’s Memorial Hermann in Houston, Texas (Sub-group B), were retrospectively reviewed. Twelve patients were excluded due to spinal surgery for something other than scoliosis or Scheuermann’s kyphosis. All 36 remaining patients underwent posterior instrumented fusions using the same surgical technique.

Quality improvement (QI) measures were prospectively evaluated to monitor radiation exposure, fluoroscopic use and accuracy of PS placement. Fourteen dosimeters (Instadose, Smyrna, GA) were placed in the operating room (OR): on the patient, surgeons, and other locations in the room. Radiation exposure (mSv) levels from the dosimeters were recorded and cumulated. Other QI data collected included diagnosis, number of instrumented levels, total number of PS, number of PS missed, OR time, cumulative fluoroscopic dose administered (mGy), total fluoroscopy time, and complications.

Accuracy of PS placement was prospectively analyzed using the technique outlined by Kim et al. describing a highly sensitive and specific method to detect a malpositioned screw [9]. The tip of the PS is evaluated after final placement under a PF shot. A tip past the midline indicates a medial breach. A tip not past the medial wall of the pedicle indicates a lateral breach. If a screw was deemed malpositioned, then it was removed or replaced and considered a “missed screw” [9]. The percent accuracy of PS placement and radiation data were compared to published literature.

### Surgical technique

**Fig. 1:**
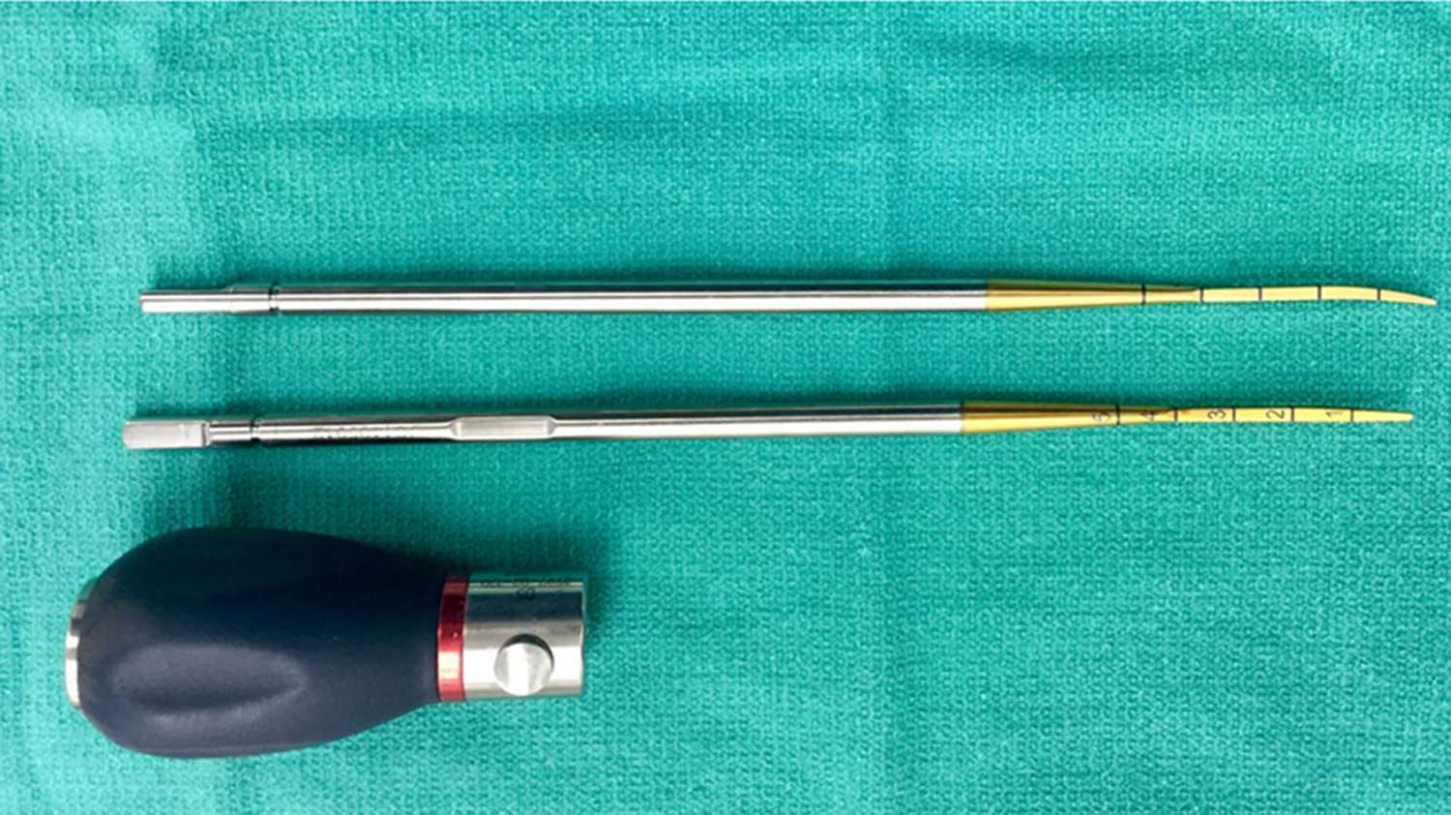
Custom-designed detachable pedicle marker and probe (DPMP) (Depuy Synthes, Raynham, MA)

**Fig. 2:**
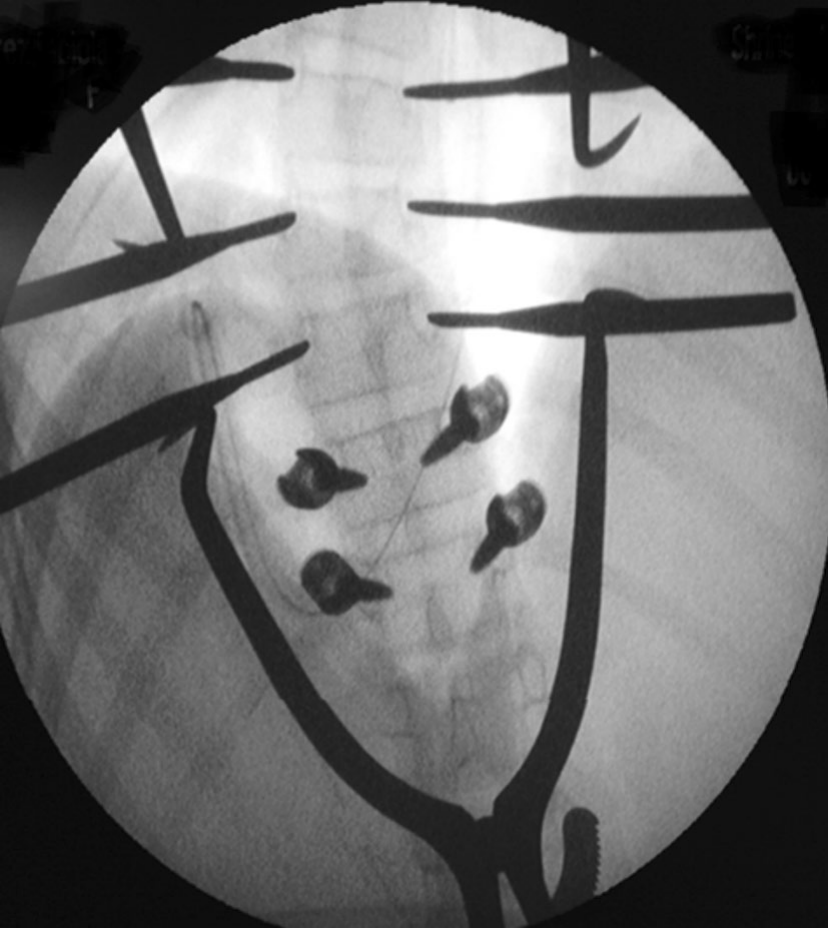
Intraoperative AP image of the vertebra with care taken to match the rotation and cant of the fluoroscopy machine with the rotation and sagittal orientation of three consecutive vertebra

The awl is used to create a starting point for a thoracic PS by identifying the osteologic right angle formed between the transverse process and the superior articular process of the vertebral body as described by Suk and Lenke [10, 11]. T10–T12 is usually instrumented first as the pedicles are typically larger and relatively neutral. A custom designed slightly curved thoracic pedicle probe with a detachable ball handle (Depuy Synthes, Raynham, MA) is tamped into the starting hole initially pointed laterally (Fig. 1). One to six of these detachable pedicle marker and probes (DPMPs) are gently tamped into a depth of 10 mm in three consecutive vertebrae. The detachable handle allows the DPMP to serve as a marker without the bulky radiopaque handle obstructing the bony landmarks. The detachable handle also allows the DPMP to stay within the starting point without toppling over. The handle is then attached and the DPMP can immediately serve as a sounding probe without interference from the other DPMP handles. Fluoroscopic assistance is then performed using a single, pulsed true AP image of the vertebrae taking care to match the rotation and cant of the machine with the rotation and sagittal orientation of three consecutive vertebrae (Fig. 2). All of our radiation technologists were experienced fluoroscopists who were taught the technique during the index cases. OR staff are asked to stand at least 6 feet away from the machine and stand behind a lead shield. The level and appropriateness of the starting point are confirmed. The base of the machine is moved cephalad to allow adjustments to the starting points. These adjustments are made as recommended by Suk et al. to assure that the DPMP starts at the 9 o’ clock position on the left pedicle and the 3 o’ clock position on the right pedicle [10]. The position of the tip of the probe should be at or lateral to the center of the pedicle. The removable ball handle is then placed on the DPMP and the probe is advanced. Probes in large-diameter pedicles (greater than 6 mm) can be advanced 20 mm as long as the tip of the probe remains parallel to the end plate and is aimed toward the center of the contralateral pedicle. The left and right probes are then medially directed and advanced to 25–30 mm in from T1 to T3, 30–35 mm from T4 to T8, and 35–40 mm from T9 to T12. The ipsilateral DPMP is removed and the contralateral DPMP is left in place to provide a visual guide to assist with tapping and screw placement. A ball-tipped probe is used for cortical breach of the pedicle or anterior cortex. The tap is then tapped to 2 mm longer than the proposed screw length. The ball-tipped probe is again used to rule out a cortical breach. If such a breach is detected, then the screw can be omitted or the DPMP can be placed in a different trajectory.

If there is no such breach, a screw 2–5 mm shorter than the depth tapped can be placed taking care to visually align the handle holding the screw with the contralateral DPMP. This technique increases the likelihood of harmonious and symmetric alignment of the screw tips. The screw handle is left on the screw, while the contralateral DPMP is used to sound the pedicle and then this PS is placed. All six screws are placed and up to six more DPMPs are placed in the mid-thoracic starting holes. A second PF shot is taken to confirm the accuracy of screw placement by re-centering the fluoroscope over the recently placed PSs. Meticulous centering of the screws under the image intensifier in perfect sagittal and coronal alignment minimizes the number of fluoroscopic pulses required, which in turn minimizes radiation exposure to the patient and staff. Accuracy of screw placement is then assessed as described by Kim et al. [9]. The machine is then moved cephalad and rotation and cant are adjusted to match the next three vertebrae with DPMPs within the starting points. A third PF shot is taken to assist with placement of these screws and six more DPMPs are placed. At this point, as few as three pulse images have been used to place up to twelve PSs and an additional six DPMPs. A fourth PF shot is taken to confirm accurate placement of the last six PS placed and then another single PF image centered over the more cephalad DPMPs is obtained to assist with placement of these PSs.

More pulsed images may be necessary to place PSs within smaller (2–5 mm) pedicles. In these cases, the probe can be advanced to a depth of 15 mm and a pulse image obtained to confirm that the tip of the probe is located between the center of the pedicle and the medial wall. If so, the probe is advanced to a depth of 20 mm and another pulse image is obtained to confirm that the tip is at or just lateral to the medial wall of the pedicle. The tip of the probe is now usually within the vertebral body; so the probe is then advanced 1–2 mm and rotated 180°, medially angulated, and advanced to the appropriate depth. PF can be used with the tap and screw advancement when deemed necessary. A PS is either omitted, or a starting point that is 2–3 mm lateral to the lateral wall of the pedicle is used for extremely small pedicles without cancellous bone. Appropriate screw length is then confirmed with lateral fluoroscopy. Post-operative CT scans were not obtained to minimize radiation exposure to patients. Our technique is not freehand with a confirmatory fluoroscopy check. It is a technique that uses fluoroscopic assistance to provide assurance, without guesswork, that the starting point of the DPMP is ideal prior to sounding the pedicle, thus decreasing anxiety and stress associated with PS placement.

### Statistical analysis

Radiation exposure (mSv), fluoroscopy time (in seconds), and cumulative fluoroscopic dose (mGy) were calculated per case. The total number of PS verified as accurately placed and “misplaced” were used to determine percent accuracy. These values were compared to historical controls in which PF with standard instruments (SI) or O-arm was used to assist and confirm PS placement.

## Results

There were 26 females and 10 males (mean age 14.6 years; range 9–21 years). The majority of subjects were diagnosed with adolescent idiopathic scoliosis (63.9%) (Tables 1 and 2). There were 11.6 ± 2.6 instrumented levels with 16 ± 6.0 PS placed. The mean time of total fluoroscopy use was 13.4 s (range 6–32.4). The mean cumulative fluoroscopic dose in patients was 3.1 mGy (range 0.2–16.4) (Table 3). The median effective dose was 0.22 mSv (range 0–7) for the patient; 0.00 mSv (range 0–0.03) for the surgeon; and 0.00 (range 0–0.05) for the OR staff. Median effective dose was significantly lower (*p* = 0.03) between the two institutions (0.04 and 0.67 mSv, respectively) (Table 4). Of the PSs placed, 561 of 576 (97.4%) were accurately placed. No neurological complications associated with misplaced screws were detected.

### Historical comparisons

Our technique using DPMPs reduced the amount of fluoroscopy to 1.2 s/level compared to the lowest previously reported 4.49 s/level reported by Su et al. using SI combined with PF to assist and confirm PS placement [12]. The current technique also reduced fluoroscopy to 0.84 s/screw compared to the lowest previously reported 7.36 s/screw reported by Haque et al. using SI combined with PF to assist and confirm PS placement [13]. Lange et al. determined the calculated effective dose from a single CT scan using the O-arm using dosimeters placed on spine models to be 3.2 ± 0.04 mSv in a small patient and 8.1 ± 0.23 mSv in a large patient [14]. An O-arm spin is required every 4–6 levels for image registration and confirmatory purposes. Small patients requiring more than 10 levels of instrumentation will, thus, require up to 3 CT scans to assist and confirm PS placement and thus receive up to 9.72 ± 0.12 mSv and large patients would receive up to 24.27 ± 0.69 mSv after 3 O-arm spins [14]. Su et al. estimated the effective dose by converting the dose length product into organ doses using coefficients and tissue weighting factors and low-dose pediatric CT scan settings on the O-arm [12]. Each patient received 1–2 scans (14 patients; mean instrumented levels, 11) and their estimated effective dose was 1.11 ± 0.17 mSv to assist with screw placement compared to 0.22 mSv using the DPMPs and PF to assist and confirm screw placement (See Table 5).

**Table 5:** Comparative studies

Study	Type of imaging	Surgical technique	# of patients	Mean # instrumented levels	Mean # of screws	Mean fluoroscopy time (s)	Radiation dose (effective dose in mSv)
This study (All)	Pulsed fluoroscopy	Freehand with fluoroscopy assist and confirmation	36 pediatrics	11.6	16	13.4	0.22
This study (Sub-group A)^a^	Pulsed fluoroscopy	Freehand with fluoroscopy assist and confirmation	20 pediatrics	11	12.5	9	0.04
This study (Sub-group B)^a^	Pulsed fluoroscopy	Freehand with fluoroscopy assist and confirmation	16 pediatrics	12.4	20.4	18.8	0.67
Hague et al. [13]	Pulsed fluoroscopy	Freehand with fluoroscopy assist and confirmation	14 pediatrics	Not available	23	167.64	1.52
Su et al. [12] (Sub-group A)	Pulsed fluoroscopy	Freehand with fluoroscopy assist and confirmation	4 pediatrics	10.5	Not available	66.5	0.452
Su et al. [12] (All)	Fluoroscopy and pulsed fluoroscopy	Combined group of Freehand with fluoroscopy assist and confirmation and Freehand with confirmation only	14 pediatrics	10.2	Not available	35.34	0.265
Su et al. [12]	CT	Stealth navigation	14 pediatrics	10.8	Not available	n/a	1.114
Lange et al. [14]	CT	Spine model	Model	9 to 10	n/a	n/a	19.44
Van de Kelft et al. [15]	CT	Stealth navigation	353 adults	5	Not available	n/a	14.58

Radiation exposure in the Van de Kelft et al. [16] articles is not expressed in units of effective dose. O’Donnel et al. [17] converted the Van de Kelft et al. [15] data using published dose-to-dose length conversion factors

^a^These study data were sub-grouped by institution. Sub-group A was Shriners Hospitals for Children; Sub-group B was Children’s Memorial Hermann in Houston, Texas. Su et al. [12] was sub-grouped by surgical technique

**Table 1:** Patients per diagnosis (*n* = 36)

Adolescent idiopathic scoliosis	23 (63.9%)
Scheuermann’s kyphosis	4 (11.1%)
Juvenile idiopathic scoliosis	2 (5.6%)
Neurogenic scoliosis	2 (5.6%)
Neuromuscular scoliosis	2 (5.6%)
Neurofibromatosis	1 (2.8%)
Congenital	1 (2.8%)
Kyphoscoliosis	1 (2.8%)

**Table 2:** Patients per Lenke classification (*n* = 25)

1A	6 (24%)
1B	4 (16%)
1C	5 (20%)
2A	2 (8%)
2B	1 (4%)
3B	1 (4%)
3C	1 (4%)
4C	1 (4%)
5A	1 (4%)
5C	2 (8%)
6B	1 (4%)

**Table 3:** Group descriptives

	All (*n* = 36)			Sub-group A (*n* = 20)			Sub-group B (*n* = 16)		
	Mean	Min	Max	Mean	Min	Max	Mean	Min	Max
# of instrumented levels	11.6 ± 2.6	6	15	11 ± 3.1	6	14	12.4 ± 1.7	10	15
Total # of pedicle screws	16 ± 6	10	27	12.5 ± 4.8	10	26	20.4 ± 4.2	11	27
Total fluoroscopy time (s)	13.4 ± 7.5	6	32.4	9 ± 4.1	6	18	18.8 ± 7.3	9.5	32.4
Cumulative fluoroscopy dose (mGy)	3.1 ± 3.4	0.2	16.4	1.7 ± 0.9	0.2	3.5	4.8 ± 4.4	0.5	16.4

These study data were sub-grouped by institution. Sub-group A: Shriners Hospitals for Children; Sub-group B: Children’s Memorial Hermann in Houston, Texas

**Table 4:** Median-estimated effective dose (mSv)

	Patient	Surgeon	Anesthesia staff	Surgical staff
All (*n* = 36)	0.22 (0–7)	0.00 (0–0.03)	0.00 (0–0.04)	0.00 (0–0.05)
Sub-group A (*n* = 20)	0.04 (0–1.68)	0.00 (0–0.03)	0.00 (0–0.04)	0.00 (0–0.05)
Sub-group B (*n* = 16)	0.67 (0.07–7)	0.00 (0–0)	0.00 (0–0)	0.00 (0–0.04)

These study data were sub-grouped by institution. Sub-group A was Shriners Hospitals for Children; Sub-group B was Children’s Memorial Hermann in Houston, Texas

In a large prospective study evaluating O-arm placement of 1922 lumbar PSs in 353 adult patients, the mean cumulative radiation dose was 10.6 ± 14.0 mGy (effective dose, 14.58 mSv) [15]. This is in sharp contrast to our cumulative radiation dose of 3.1 mGy. Of further note, our median effective dose to the surgeon and OR staff was 0.0 mSv (range 0–0.03) per case as compared to 1.52 mSv found in Haque et al.; which in their study translated to 13.49 mSv of exposure per year, prompting a call for methods to lower such exposure [13]. Our technique would be an answer to this call.

Multiple studies have been done to assess PS accuracy in procedures using O-arm. The largest appears to be that of Van de Kelft et al., who observed an accuracy of 97.5% in the placement of 1922 lumbar PSs [15]. This is similar to the 97.9% found by Rajasekaran et al. in the placement of 242 thoracic PSs, and in particular, our 97.4% accuracy rate associated with PF [16].

## Discussion

Previous studies have shown that fluoroscopy with SI or O-arm is useful for safe PS placement; however, this level of assist and confirmation results in significant radiation exposure to the surgeon, staff, and patient [12–15]. Our technique using DPMPs and fluoroscopy to assist and confirm PS placement can achieve results similar to techniques using O-arm or fluoroscopy with SI but with considerably less radiation exposure.

Studies supporting O-arm focus on radiation exposure to surgeons, rather than patients [16, 18–21]. While O-arm may reduce radiation exposure to surgeons, it drastically increases radiation exposure to patients, whose health and well-being is the direct responsibility of the surgeon. It may be prudent to demonstrate higher quality evidence showing superiority of O-arm to justify exposing patients to substantially higher levels of radiation, especially patients with immature breast and gonadal tissue. While O-arm has benefits in specific scenarios, we should be mindful of the amount of radiation exposure to our patients and use it judiciously.

Our technique is a hybrid and refinement of practices described by Suk, Lenke and Shufflebarger [10, 11, 13]. We use the anatomic landmarks described by Lenke [11] to place specially designed pedicle probes to identify safe starting points in a similar fashion described by Suk [10]. We do not use AP and lateral X-rays to assist safe marker entry points as described in Suk’s original freehand technique, which in turn reduces radiation exposure [10]. Using one fluoroscopic pulse to place up to six PSs over three levels decreases fluoroscopy time compared to Shufflebarger’s technique, where multiple images are used to assist and confirm placement of two PSs at one level [13]. Our technique does expose the patient to more radiation than the free hand technique described by Lenke. However, our technique provides increased assurance that the starting point and trajectory of the DPMPs will safely cannulate the pedicle compared to Lenke’s technique which is based on anatomical landmarks alone. The assurance of a safe starting point decreases the anxiety and stress associated with PS placement.

There are several limitations to our study. Although the technique to confirm accurate placement of PSs described by Kim et al. has a high-level specificity and validity, its sensitivity and positive predictive value are lower than those using post-operative CT scans [9]. However, we do not believe that the possible benefits of a post-operative CT scan to confirm accurate screw placement outweigh the potential risks of additional radiation exposure especially when AP and lateral images have verified accurate screw placement and the patients are asymptomatic. Also, the dose will vary from one machine to another and the effective dose measured by dosimeters may not be comparable to calculated doses based on coefficients and tissue weighting. Lastly, though we assume that more radiation exposure is suboptimal, we do not know the long-term effects of increased radiation exposure from standard fluoroscopy and O-arm.

Our spinal instrumentation technique using pulsed fluoroscopic guidance with DPMPs is a viable alternative to fluoroscopy with SI and O-arm as it has comparable accuracy with lower radiation exposure to the patient, surgeon and OR staff.

## Key points

- Our technique of posterior spinal instrumentation using DPMPs and pulsed fluoroscopy to assist and confirm pedicle screw placement can achieve results similar to techniques using O-arm or fluoroscopy using standard instrumentation but with considerably less radiation exposure.
- Pedicle screw placement using DPMPs and pulsed fluoroscopy reduced the effective dose of radiation exposure to the patient by 80% (0.22 mSv vs. 1.11 mSv) when compared to low-dose O-arm.
- Using DPMPs and pulsed fluoroscopy reduced the amount of fluoroscopy used to 1.2 s/level compared to the lowest previously reported 4.49 s/level reported by Su et al. using standard freehand instruments combined with pulsed fluoroscopy to assist and confirm pedicle screw placement.
- The current technique also reduced fluoroscopy use to 0.84 s/screw compared to the lowest previously reported 7.36 s/screw reported by Haque et al. using standard freehand instruments combined with pulsed fluoroscopy to assist and confirm pedicle screw placement.
